# Predicting declines in physical function in persons with multiple chronic medical conditions: What we can learn from the medical problem list

**DOI:** 10.1186/1477-7525-2-47

**Published:** 2004-09-07

**Authors:** Elizabeth A Bayliss, Martha S Bayliss, John E Ware, John F Steiner

**Affiliations:** 1 Department of Family Medicine, University of Colorado Health Sciences Center, Denver, CO, USA; 2 Kaiser Permanente, PO Box 378066, 80237-8066 Denver, CO, USA; 3 Quality Metric, Inc., Lincoln, RI, USA; 4 Department of Internal Medicine, University of Colorado Health Sciences Center, Denver, CO, USA

**Keywords:** comorbidity, physical functioning, quality of life, SF-36 Health Survey

## Abstract

**Background:**

Primary care physicians are caring for increasing numbers of persons with comorbid chronic illness. Longitudinal information on health outcomes associated with specific chronic conditions may be particularly relevant in caring for these populations. Our objective was to assess the effect of certain comorbid conditions on physical well being over time in a population of persons with chronic medical conditions; and to compare these effects to that of hypertension alone.

**Methods:**

We conducted a secondary analysis of 4-year longitudinal data from the Medical Outcomes Study. A heterogeneous population of 1574 patients with either hypertension alone (referent) or one or more of the following conditions: diabetes, coronary artery disease, congestive heart failure, respiratory illness, musculoskeletal conditions and/or depression were recruited from primary and specialty (endocrinology, cardiology or mental health) practices within HMO and fee-for-service settings in three U.S. cities. We measured categorical change (worse vs. same/better) in the SF-36^® ^Health Survey physical component summary score (PCS) over 4 years. We used logistic regression analysis to determine significant differences in longitudinal change in PCS between patients with hypertension alone and those with other comorbid conditions and linear regression analysis to assess the contribution of the explanatory variables.

**Results:**

Specific diagnoses of CHF, diabetes and/or chronic respiratory disease; or 4 or more chronic conditions, were predictive of a clinically significant decline in PCS.

**Conclusions:**

Clinical recognition of these specific chronic conditions or 4 or more of a list of chronic conditions may provide an opportunity for proactive clinical decision making to maximize physical functioning in these populations.

## Background

Comorbid chronic diseases are increasingly recognized as a significant factor in declining health. Of the 125 million Americans with chronic diseases, 48% are estimated to have at least one comorbidity, and 62% of persons over the age of 65 have two or more chronic illnesses.[1,2] Approximately 25% of persons with chronic illness have some limitation in activity and the percent of persons with disability increases with increasing numbers of coexisting conditions.[2,3] As the population in the United States ages, the number of persons with comorbid chronic disease will increase substantially. Primary care physicians will both provide and coordinate much of the care for this population [4].

Primary care for persons with chronic medical conditions differs from specialist care for these same conditions in the need to see both the forest and the trees: to address disease-specific issues and outcomes in the context of both coexisting medical conditions and the patient's psychosocial environment. To this end, practice guidelines developed from randomized controlled trials with strict inclusion criteria may not generalize well to the heterogeneity of primary care practice or the complex individual patient.

In the environment of competing demands that characterizes medicine in general, and primary care in particular; information on health outcomes that can be inferred from the medical record problem list may be particularly relevant in clinical decision making for persons with multiple chronic conditions. We chose to investigate the health outcome of physical well being for several reasons: It is relevant to both clinical and quality-of-life decision making; and previous findings have demonstrated that different combinations of chronic medical conditions have been shown to be associated with type and/or severity of disability [5,6].

Longitudinal studies suggest that the cumulative effect of comorbid conditions is not simply additive: certain combinations of diseases may have a greater effect on outcomes than others. Combinations of diabetes plus obesity or heart disease [7], and arthritis plus diabetes, pulmonary disease or obesity [8] were significantly more detrimental to measures of health outcomes than either condition alone or in combination with other comorbidities. In a review of multiple longitudinal studies on comorbidity, Gijsen et al. found comorbidity to be a predictor of higher mortality, worse functional status, decreased quality of life, and increased health care utilization [9]. It is impractical for the primary care physician to maintain an awareness of specific combinations of chronic conditions that may characterize patients at risk for functional decline. However recognizing certain chronic medical conditions as potential 'red flags' for further investigation would be useful.

We hypothesized that certain chronic diseases that often occur as comorbidities may have a greater impact than others on functional status outcomes over time. To explore this question, we analyzed data from the Medical Outcomes Study (MOS), a longitudinal study focusing on the care and medical outcomes of patients with specific common chronic conditions [10]. The MOS data have been previously used to study comparative effects of chronic conditions on physical well being over time [11-14]. These investigations have included analyses of the effects of anxiety disorder, varying levels of physical activity, and depression on health outcomes in the context of multiple chronic diseases.[12,15,16] We used this important data set to further explore the complexities of long-term health outcomes in a heterogeneous group of patients with comorbid chronic disease.

Our study used MOS data to investigate the relative effect of six different common chronic conditions (diabetes, coronary artery disease (CAD), congestive heart failure (CHF), chronic respiratory disease, musculoskeletal disease and depression) on measures of physical well being over the course of four years in patients with comorbidities. This data base is comprised of information from respondents with certain chronic medical conditions. There is no information on 'healthy' respondents without any chronic conditions. Therefore we examined the relative effects of selected conditions relative to hypertension alone. We examined the presence of a specified level of decline in physical well being that had been verified to be clinically significant, rather than a change in the PCS score that might be statistically significant but of limited practical importance to patients. In addition, we analyzed the effect of specific diagnoses in the context of the total disease burden in an effort to identify possible 'sentinel' conditions that may specifically contribute to functional decline for patients with multiple comorbidities.

## Methods

### Study design

The MOS was a four-year observational study that included assessment of health outcomes of chronically ill patients. Details of the study including design, sampling, site selection and clinician recruitment have been previously published[10,16-18]

### Study setting

MOS study sites were selected from three cities (Boston, MA, Chicago, IL and Los Angeles, CA), from both primary care (family practice and internal medicine) and specialty (endocrinology, cardiology and mental health) practices, and from both managed care and fee-for-service payment plans.

### Sample and data collection

The original study sample consisted of patients with one or more of five chronic "tracer" conditions (hypertension, adult onset diabetes, myocardial infarction within the past six months, congestive heart failure or depression) approached during a visit with an MOS clinician during a two-week period in 1986. Of the 28,257 patients originally approached, 20,232 agreed to participate. From this group, patients were selected for follow up on the basis of diagnosis and completion of baseline data collection, as described elsewhere [18,19]. Of the 3589 patients selected for follow-up, 2708 completed a baseline assessment, and 2235 were randomly selected for follow-up. Four-year follow-up data were obtained for 1574 of these 2235 (70%). For the current study, the chronic conditions of interest (diabetes, CAD, CHF, chronic respiratory disease, musculoskeletal conditions, and depression) were defined by combining the original tracer diagnoses with additional diagnoses determined by a structured medical history interview conducted by a trained clinician [17]. For example our category of CAD consists of persons with the original tracer condition of myocardial infarction within the past 6 months plus those with a history of angina, current symptoms of angina, and myocardial infarction more than one year ago. If information about a condition was missing, an independently derived probability of each diagnosis was substituted if the probability was at least 90%. The components of each of the main diagnoses are listed in Table 1. We chose to analyze these conditions based on their high prevalence as well as frequent assessment in the literature on comorbidity and chronic disease management.

**Table 1:** Components of main disease categories

**Main Disease**	**Number (percent)^a^**
**Diabetes**	**359**
Type 2 diabetes mellitus	319 (88.9)
Type 1 diabetes mellitus	40 (11.1)
**CAD**	**425**
Myocardial infarction within past 6 months	76 (17.9)
History of angina	104 (24.5)
Current angina	233 (54.8)
Myocardial infarction more than one year ago	135 (31.8)
**CHF**	**159**
**Respiratory disease**	**133**
Asthma	42 (31.6)
Chronic obstructive pulmonary disease	95 (71.4)
Other lung disease	23 (17.3)
**Musculoskeletal disease**	**684**
Back pain	446 (65.2)
Musculoskeletal complaints	277 (40.5)
Hip impairment	55 (8.0)
Osteoarthritis	145 (21.2)
Rheumatoid arthritis	30 (4.4)
**Depression**	**555**
Diagnosed depression	260 (46.8)
Symptoms of depression	295 (53.2)

a) May sum to more than 100% due to coexisting conditions.

The final sample included patients who had complete baseline and four-year follow-up information (including deaths), had completed a medical history questionnaire, and had definitive diagnostic information on their original tracer condition [17]. Patients from the longitudinal sample who were lost to follow-up did not have significant differences in initial health status from those who remained in the sample, however patients lost to follow-up tended to be younger and had lower income than those remaining [17].

### Outcome measures

We analyzed categorical change (worse versus same/better) in SF-36^® ^Health Survey (SF-36) physical component summary (PCS) scores over four years. We chose this dichotomous outcome to emphasize the clinical importance of anticipating functional decline in patients with chronic medical conditions. Categorical change was defined as a decrease of 6.5 or more points in PCS. This was based on standards for PCS scores in which a change of 6.5 points is outside the 95% confidence interval for PCS scores. Longitudinal change norms for PCS classify patients with +/- two standard errors of measurement (SEM) as "better" or "worse" and those within two SEM as "staying the same" [17,20]. Changes of 6.5 points or more in PCS over time are clinically significant and correlate with changes in health and mortality [17,20]. We also assessed linear change in PCS scores over the four-year period.

### Statistical analysis

Based on published sample sizes specifically calculated to detect differences in PCS between two groups using repeated measures over time, our sample size was adequate to detect a difference of five points in PCS with 80% power (alpha = 0.05, two tailed test with an intertemporal correlation of 0.70) [20]. As these published sample size calculations were designed to detect a slightly smaller difference in PCS than we chose to examine (5.0 versus 6.5 points), our sample sizes should be more than adequate.

We used logistic regression to analyze the independent effect of each main chronic disease on categorical change (worse vs. same/better) in PCS relative to hypertension alone adjusting for the effect of the other main diseases. Logistic regression was again used to assess change in PCS over four years in patients with one, two, three or four or more of the main chronic conditions relative to those with hypertension alone.

Due to the selection criteria for the original MOS study [10], the MOS data set does not include 'healthy' participants without any chronic conditions. Therefore we used persons with hypertension alone, but none of the other main chronic conditions, as the referent group for the analysis. In one analysis, hypertension alone had an effect on PCS that was comparable to the effect of aging in a 'healthy' population.[21,22] However another longitudinal analysis has shown slightly increased odds of a decline in health status over 2 years in patients with hypertension alone relative to those with no chronic conditions. This effect diminished with age [7].

We completed separate regressions to determine the effect on PCS due to each of the main chronic conditions of interest. In these models, the study population was divided into those with hypertension alone (referent group), those with the main chronic disease of interest (with and without other conditions), and those with any other of the main chronic conditions *other than *the condition of interest. Using similar modeling, linear regression analysis was used to assess the relative contributions of the explanatory variables. There were no significant interactions between each of the main conditions and the total number of conditions. As age has been shown to be associated with functional outcomes in persons with comorbidities [23], we assessed categorical change in PCS by number of comorbid conditions relative to hypertension alone for older and younger age groups (under 65 years vs. 65 and over). Four-year change in PCS relative to hypertension was comparable in both age groups, therefore the final analysis was not stratified by age.

Analyses were additionally adjusted for age, a count from a list of 16 additional chronic conditions (in addition to adjustments for main diseases as mentioned above), poverty level, gender, race, educational level, employment status, and marital status. Subjects who died during the course of the study were assigned a four-year PCS score of zero and included in the 'worse' category. Assignment of a zero PCS sore to participants who died during the course of the study has been discussed by Diehr et al. as a reasonable approach for analyses in which a decline in health is the outcome of interest [17,24]. Failure to incorporate these subjects could substantially bias the results by limiting the assessment of outcomes to 'healthier' subjects. To partially account for level of physical well being at baseline, the analyses also adjusted for starting PCS score relative to age/gender norms.

## Results

Of a total of 1574 subjects, 281 individuals carried a diagnosis of hypertension exclusive of any of the other major comorbid conditions, and were defined as the referent population for this analysis. (As participants in the original MOS study were selected on the basis of chronic medical diagnoses, the study population did not include a referent 'disease-free' population.) The remaining 1293 subjects had either one or more of the six comorbid conditions of diabetes, CAD, CHF, respiratory disease, musculoskeletal disease and depression with or without hypertension. In this heterogeneous population, subjects with the main conditions of interest had, on average, 1.8 of the main conditions and 0.8 from a list of 16 additional conditions. Referent subjects with hypertension and no other main conditions of interest had 0.3 additional conditions. The majority of all respondents had starting PCS scores within 1 standard deviation of age/gender norms, with an additional 10% above and 26% below age gender norms. Table 2 describes the characteristics of the study population.

**Table 2:** Characteristics of study population

**N**	**1574**
Age (mean) +/- SD	57.6 +/- 15.4
Male	41.3%
Married	58.3%
Employed	46.4%
At or below 200% of poverty level	19.3%
White (vs. non-white)	82.5%
**Education**	
Education less than high school	14.6%
High School graduate	28.5%
Greater than high school	28.5%
College graduate	12.1%
Greater than college	16.3%
Mean number of main diseases*	1.5
Hypertension alone (referent group)	0
Remaining subjects	1.8
Mean number of additional diseases**	0.7
Hypertension alone (referent group)	0.3
Remaining subjects	0.8
**PCS Scores**	
Baseline PCS score > 1 standard deviation above age/gender norms	10.0%
Within 1 standard deviation of age/gender norms	63.7%
>1 standard deviation below age/gender norms	17.9%
>2 standard deviation below age/gender norms	8.5%

* Diabetes, coronary artery disease, congestive heart failure, musculoskeletal disease, respiratory disease and depression.

** From a list of 16 additional conditions.

Subjects with CHF, diabetes or chronic respiratory disease had increased odds of a clinically significant decline in PCS over 4 years. These odds ratios (confidence intervals) were 2.9 (1.7, 5.0), 2.1 (1.5, 2.9) and 1.7 (1.1, 2.8) respectively (Table 3). Subjects with diagnoses of CAD, musculoskeletal disease, or depression did not show a significant change in physical well being over 4 years relative to the referent population. The effects of these main conditions on physical well being over 4 years were confirmed by the linear model in which subjects with diagnoses of CHF, diabetes or respiratory disease had adjusted 4-year declines in PCS scores of -10.0, -3.2 and -3.1 points (p < = 0.05 for all).

**Table 3:** Adjusted odds of a decline in PCS attributable to presence vs. absence of each main chronic disease^a ^(Total N = 1574)

**Disease**	**N^b^**	**Adjusted odds ratio**
Hypertension	281	1.0
Diabetes	249	**2.1 (1.5, 2.9)**
Coronary Artery Disease	364	1.1 (0.8, 1.5)
Congestive Heart Failure	137	**2.9 (1.7, 5.0)**
Respiratory Disease	125	**1.7 (1.1, 2.8)**
Musculoskeletal Disease	514	0.9 (0.7, 1.2)
Depression	319	1.3 (0.9, 1.8)

a) Adjusted for number of main conditions, number of additional comorbid conditions, poverty level, gender, race, educational level, employment status, marital status.

b) Sum to more than 100% due to coexisting conditions

An absolute decrease in PCS of 6.5 points *per subject *was used as criteria for a *clinically *significant decline in PCS over time based on previous analyses of the MOS data and on validation of the SF-36^® ^survey instrument [17]. The linear regression model presents changes in PCS for the *population *of subjects adjusted for characteristics of that population. Therefore the statistically significant changes in PCS scores over time resulting from the linear regression analysis may not necessarily be greater than 6.5 points

**Figure 1:**
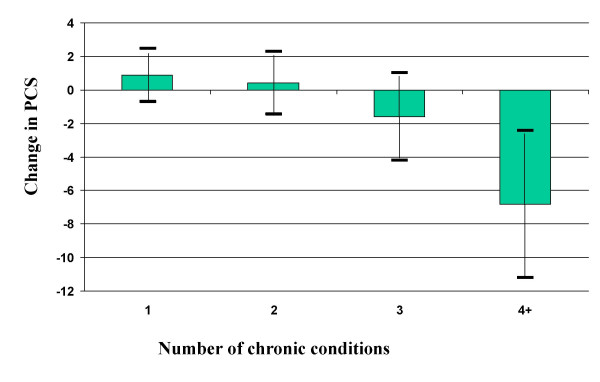
Change in PCS relative to hypertension alone by number of main chronic diseases.

Little increase in the odds of functional decline was evident in individuals with 1,2, or 3 of the main chronic conditions. However having 4 or more of these conditions predicted a decline in PCS (OR 2.8; CI 1.3, 5.9) (Table 4 and Figure 1). The effect on PCS of the number of chronic diseases was similar in older and younger age groups.

**Table 4:** Number of main chronic conditions as predictors of a decline in physical well being over four years

**Number of main chronic conditions**	**N**	**Adjusted odds of a decine in PCS^ab ^(N = 1574)**
Hypertension alone	281	1.0
One main chronic disease^c^	607	1.1 (0.8, 1.4)
Two main chronic diseases^c^	423	1.2 (0.8, 1.7)
Three main chronic diseases^c^	197	1.4 (0.9, 2.2)
Four or more chronic diseases^c^	66	**2.8 (1.3, 5.9)**

a) Adjusted for age, number of additional comorbid conditions, poverty level, gender, race, educational level, employment status, marital status.

b) Number of main chronic diseases as a predictor of a decrease in PCS is statistically significant at p < = .05

c) Subjects with one, two, three or four or more of the following: DM, CAD, CHF, musculoskeletal disease, respiratory disease, or depression. May also include hypertension.

## Discussion

Physical well being is particularly relevant for persons with chronic conditions and the clinicians who care for them. Declines in physical well being may have significant social, emotional and economic repercussions, as they correlate with job loss, high health care utilization and increased mortality [20]. Based on this analysis of a heterogeneous population from the MOS; persons with CHF, diabetes and/or chronic respiratory disease are at particular risk. Primary care providers are in an ideal position to help prevent, delay or proactively manage potential declines in physical functioning for these patients.

In this analysis of change in physical well being over time in persons with a variety of chronic medical conditions, we found that specific diagnoses of CHF, diabetes and/or chronic respiratory disease; or the presence of 4 or more chronic conditions, were predictive of a clinically significant decline in PCS. We hypothesize that our findings reflect the different clinical courses of each of these conditions as well as the varying potential for therapeutic interventions in each case. The natural history of CHF, diabetes and some respiratory disease is progressive. Treatment of these conditions is aimed at optimizing long-term health outcomes. Ongoing management involves self-care that is replete with complex concepts and tasks. In contrast, management of CAD and musculoskeletal disease may include the potential for surgical intervention and protocol-driven rehabilitation programs. While some sub-populations of patients with these latter conditions may develop increasing disability, others may experience significant improvement in physical well being over time. For example, increases in PCS scores associated with hip replacement and therapy for low back pain can be in the range of 9.5 and 7.6 respectively [20]. We have no specific information on such interventions in our study population and therefore were unable to incorporate treatment interventions into our analysis.

Disease management programs have been successful in improving health outcomes for persons with these and other chronic conditions [25,26]. However these programs are often disease specific [4,27,28]. As this study population illustrates, many chronic conditions do not occur in isolation. This may make disease-specific programs less beneficial for many patients. Some components of successful disease-management programs that are particularly relevant to persons managing multiple medical conditions include: guidance in problem solving, decision making, confidence building, self-management support, and systematic support of the disease management process [29-31].

In considering the issue of functional decline, any 'clinically significant change' over time is not only a function the starting and ending levels of functional status. It also is determined by the individual for whom the change has meaning, the instrument used to assess the change, and population norms that provide the context for the observed change [32,33]. Population-based studies (including ours) address parts of this equation, but do not address the most important dependent variable in the equation: the implications of a change in function to the individual. It is up to the provider and the patient to interpret the 'data' in the context of the individual and consider any subsequent recommendations in that same context.

Our findings on depression deserve special mention. Depression in particular and mental health in general are well known to affect the management and outcomes of several chronic medical conditions [34-36]. Furthermore there is a high prevalence of coexisting depression in persons with chronic disease [37]. In this analysis, we found that depression did not predict a decline in physical functioning over time. We hypothesize that this is partly due to the natural history of the disease: Depression has a waxing and waning course, and disease symptoms that are subject to significant environmental and social effects. Therefore the effect of depression on physical functioning in this patient population may have varied significantly within the population over time. Our findings are also consistent with previous findings on depression in this data set: When MOS subjects with depression were followed over two years [12], they were noted to have similar or improved scores of physical functioning at the end of two years relative to those at baseline. It is possible that this trend continued in our sub-sample and accounted for the non-significant change in PCS score for subjects with depression as a comorbid condition.

The study of comorbidity is, by definition, the study of inter-relationships: between different diseases and between diseases and age or other health-related sociodemographic variables. Caring for persons with chronic illness is, similarly, the care of heterogeneous populations of individuals with multidimensional medical, psychological and social issues. The heterogeneity of this study population may be relevant to the provider willing to sacrifice some internal validity in hopes of generalizing the findings to his or her patient population.

## Limitations

Our sample size precluded stratification to investigate the relative contributions of different medical conditions to physical well being in sub-populations defined by specific combinations of conditions, smaller age groups, socioeconomic status or other demographic criteria that might further clarify the interactive nature of the comorbid chronic disease process. It is likely that the effect of CHF, diabetes, chronic respiratory disease or other conditions on measures of health related quality of life differs within different subpopulations. Specifically, vulnerable populations may be more at risk of poor health outcomes due to socioeconomic status or process of care factors than specific disease states [17,38]. In addition, there may be additional psychological and sociodemographic factors (e.g. levels of self-efficacy and social support systems) that we were unable to incorporate in our model, which affect outcomes in persons with comorbid conditions. In the original MOS cross-sectional analysis of functional status and well being, most of the variance in outcomes measured (including PCS scores) was not attributable to the diseases studied the same is true of our longitudinal analysis [16].

Due to the nature of the MOS data base, we compared the effect of selected chronic conditions on physical well being to a referent group with hypertension alone. While the effect of hypertension alone on physical well being over 4 years may be minimal ; it is possible that there is a synergistic effect between hypertension and certain conditions such as CAD and CHF on physical well being over time. If so, the effect of CAD or CHF on PCS scores for persons with these conditions may have been slightly magnified. It is unlikely that this bias would change the overall significance of the results of our analysis.

We were unable to directly account for severity of illness for all main chronic conditions either at baseline or follow-up. However statistical adjustments for starting PCS score relative to age/gender norms and the inclusion of patients who died in the final analysis should indirectly account for some degree of severity of illness throughout the study population.

As in all investigations, the conclusions reflect the population studied: primarily Caucasian, with a majority above 200% of the federal poverty level, relatively well educated and specifically selected for inclusion on the basis of certain medical diagnoses. Results from this study may not be generalizable to populations with different demographic characteristics and different constellations of comorbid conditions.

## Conclusions

This analysis suggests that long term physical well being in persons with multiple chronic diseases is a function of both the number and type of medical conditions. Relative to persons with hypertension alone, those who carry diagnoses of CHF, diabetes and chronic respiratory disease have an increased risk of a decline in physical well being over 4 years. The presence of CAD, musculoskeletal disease or depression does not predict a similar decline.

For the primary care physician for whom 'comorbidity' implies an exponentially increasing ratio of items on the problem list to available time during the office visit, the specific diagnoses of CHF, diabetes and chronic respiratory disease may serve as triggers for management decisions. Clinicians who care for patients with these common conditions should be alert to the possibility that a proactive approach incorporating generalizable principles of disease management may either attenuate this loss of function or help the patient and family anticipate future needs.

## Authors' contributions

EB participated in the design of the investigation, performed analysis and drafted the manuscript. MB participated in the design of the investigation, performed additional analysis, data management, and manuscript revision. JW supervised the original data collection and MOS investigations and assisted in manuscript revision. JS participated in the design of the investigation, advised on the analysis and assisted in manuscript revision. All authors read and approved the final manuscript.
